# Germline mutations of 4567 patients with hereditary breast-ovarian cancer spectrum in Thailand

**DOI:** 10.1038/s41525-024-00400-4

**Published:** 2024-02-14

**Authors:** Chalermkiat Kansuttiviwat, Pongtawat Lertwilaiwittaya, Ekkapong Roothumnong, Panee Nakthong, Peerawat Dungort, Chutima Meesamarnpong, Warisara Tansa-Nga, Khontawan Pongsuktavorn, Supakit Wiboonthanasarn, Warunya Tititumjariya, Nannipa Phuphuripan, Chittapat Lertbussarakam, Jantanee Wattanarangsan, Jiraporn Sritun, Kittiporn Punuch, Jirayu Kammarabutr, Pornthira Mutirangura, Wanna Thongnoppakhun, Chanin Limwongse, Manop Pithukpakorn

**Affiliations:** 1 Division of Medical Genetics, Department of Medicine, Faculty of Medicine Siriraj Hospital, Mahidol University, Bangkok, Thailand; 2 Siriraj Genomics, Faculty of Medicine Siriraj Hospital, Mahidol University, Bangkok, Thailand; 3 University of Alabama at Birmingham, Birmingham, AL USA; 4 Department of Medicine, University of Minnesota Medical School, Minneapolis, MN USA

**Keywords:** Molecular medicine, Genetic testing

## Abstract

Multi-gene panel testing has led to the detection of pathogenic/likely pathogenic (P/LP) variants in many cancer susceptibility genes in patients with breast-ovarian cancer spectrum. However, the clinical and genomic data of Asian populations, including Thai cancer patients, was underrepresented, and the clinical significance of multi-gene panel testing in Thailand remains undetermined. In this study, we collected the clinical and genetic data from 4567 Thai patients with cancer in the hereditary breast-ovarian cancer (HBOC) spectrum who underwent multi-gene panel testing. Six hundred and ten individuals (13.4%) had germline P/LP variants. Detection rates of germline P/LP variants in breast, ovarian, pancreatic, and prostate cancer were 11.8%, 19.8%, 14.0%, and 7.1%, respectively. Non-*BRCA* gene mutations accounted for 35% of patients with germline P/LP variants. *ATM* was the most common non-*BRCA* gene mutation. Four hundred and thirty-two breast cancer patients with germline P/LP variants (80.4%) met the current NCCN genetic testing criteria. The most common indication was early-onset breast cancer. Ten patients harbored double pathogenic variants in this cohort. Our result showed that a significant proportion of non-*BRCA* P/LP variants were identified in patients with HBOC-related cancers. These findings support the benefit of multi-gene panel testing for inherited cancer susceptibility among Thai HBOC patients. Some modifications of the testing policy may be appropriate for implementation in diverse populations.

## Introduction

Breast cancer is the most common malignancy worldwide and has contributed to a significant impact on global cancer-related deaths^1,2^. Hereditary cancer syndromes accounted for ~5–10% of all cancer patients^3,4^. Breast cancer is among the most common cancers with genetic susceptibility, with *BRCA1* and *BRCA2* regarded as the most identified genes^5^. Besides *BRCA1/2*, many pathogenic/likely pathogenic (P/LP) variants in high and moderate penetrance genes for breast-ovarian cancer, including *TP53, CDH1, PALB2, STK11, PTEN, CHEK2, ATM, BARD1, BRIP1*, and *RAD51D*, were increasingly identified after the advent of next-generation sequencing (NGS)-based testing^5–7^. Many studies have shown that non-*BRCA* cancer susceptibility genes contribute to increased breast and other cancer risk^8,9^. With expanded access to comprehensive and lower-cost NGS-based multi-gene panels, more patients and families with cancer-predisposing gene mutations could be found, leading to proper screening, early detection, and cancer prevention in at-risk individuals^10^.

The prevalence of *BRCA1* and *BRCA2* pathogenic variants in breast and other related cancers in various populations and clinical management guidelines of affected individuals were well established, and similar data of non-*BRCA* breast cancer susceptibility genes were also increasingly published. Though most prevalence data of non-*BRCA* pathogenic variants in hereditary breast cancer were from Western countries, several studies in the Asian population have shown the significant detection rate and distribution of pathogenic variants in non-*BRCA* genes in different countries^11–15^. A study by Su Y et al.^11^ in China identified 12.2% of high-risk breast cancer patients who harbored pathogenic variants in non-*BRCA* genes. Another study in India showed that 15.1% of germline pathogenic variants in breast-ovarian cancer patients were from non-*BRCA* genes^15^. These findings have highlighted the importance of expanding genetic tests beyond *BRCA1* and *BRCA2* for breast and ovarian cancer patients.

Germline *BRCA1* and *BRCA2* testing for clinically indicated breast and ovarian cancer patients has proven cost-effective worldwide and in Thailand^16–18^. The test is covered by health insurance and integrated into many national healthcare systems worldwide. In Thailand, the universal reimbursement of germline *BRCA1* and *BRCA2* genetic testing for breast cancer patients has been approved since 2022. However, recommendations regarding genetic testing other than *BRCA1* and *BRCA2* continue to vary between countries, and the data and testing policy of non-*BRCA* genes among Thai patients with the hereditary breast-ovarian cancer (HBOC) spectrum remains insufficient. Therefore, we aim to identify the prevalence of hereditary cancer in Thai patients with HBOC spectrum and the contribution of non-*BRCA* breast cancer susceptibility genes detected by multi-gene panel testing. We also aim to demonstrate the clinical phenotypes of patients with identified pathogenic/likely pathogenic (P/LP) variants and compare those clinical phenotypes to the current testing criteria from the U.S. National Comprehensive Cancer Network (NCCN) clinical practice guidelines.

## Results

**Fig. 1:**
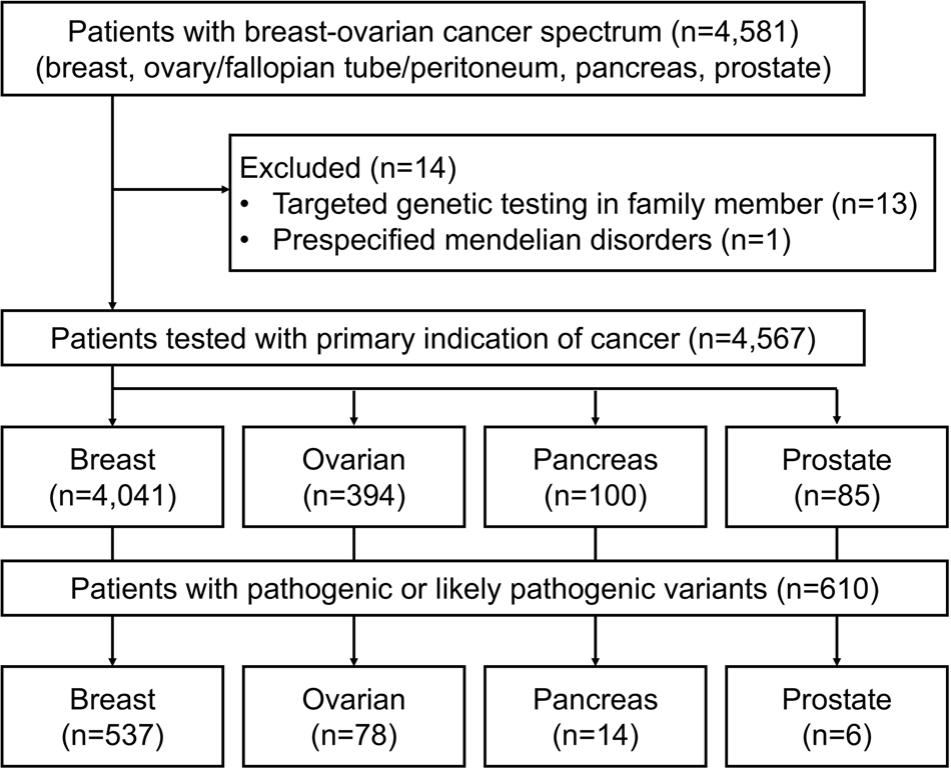
Diagram of the study cohort.

There were 4567 patients with HBOC spectrum tested with multi-gene panel testing due to HBOC-related cancers, consisting of 4041 breast, 394 ovarian (including fallopian tube and primary peritoneal cancer), 100 pancreatic, and 85 prostate cancer patients (Fig. 1). Six hundred and ten patients (13.4%) had at least one P/LP variant. Mutations in *BRCA1, BRCA2*, and non-*BRCA* cancer susceptibility genes accounted for 34.9% (*n* = 213), 31.6% (*n* = 193), and 35.1% (*n* = 214), respectively. The detection rate and distribution of P/LP variants in patients with each type of cancer are demonstrated in Table 1.

**Table 1:** Detection rate of P/LP variants and distribution of variants in *BRCA1/2* and non-*BRCA* genes classified to different types of HBOC-related cancers

	Breast		Ovary		Pancreas		Prostate	
Patients with P/LP variants/total patients tested (% detection rate)	491/4041 (11.8%)		78/394 (19.8%)		14/100 (14.0%)		6/85 (7.1%)	
*BRCA* genes								
- BRCA1	178 (36.3%)		40 (51.3%)		3 (21.4%)		1 (16.7%)	
- BRCA2	178 (36.3%)		17 (21.8%)		3 (21.4%)		2 (33.3%)	
Non-*BRCA* genes associated with each cancer type	*PALB2* *TP53* *CDH1* *PTEN* *ATM* *CHEK2* *NF1* *BARD1* *RAD51C* *RAD51D*	36 24 3 2 37 5 6 13 5 8	*PALB2* *ATM* *RAD51D* *MLH1* *MSH2* *PMS2*	2 6 3 3 5 3	*ATM* *CDKN2A* *BARD1* *RAD51C*	5 1 1 2	*MLH1* *MSH6* *RAD50*	1 1 1
Total	139 (28.3%)		22 (28.2%)		8 (57.1%)		3 (50.0%)	
Others cancer susceptibility genes identified	*RAD50* *BRIP1* *MLH1* *MSH2* *MSH6* *PMS2* *NBN3* *XRCC2* *CDKN2A*	16 8 3 6 2 7 3 2 1	*ERCC2*	1				
Total		48		1				

**Fig. 2:**
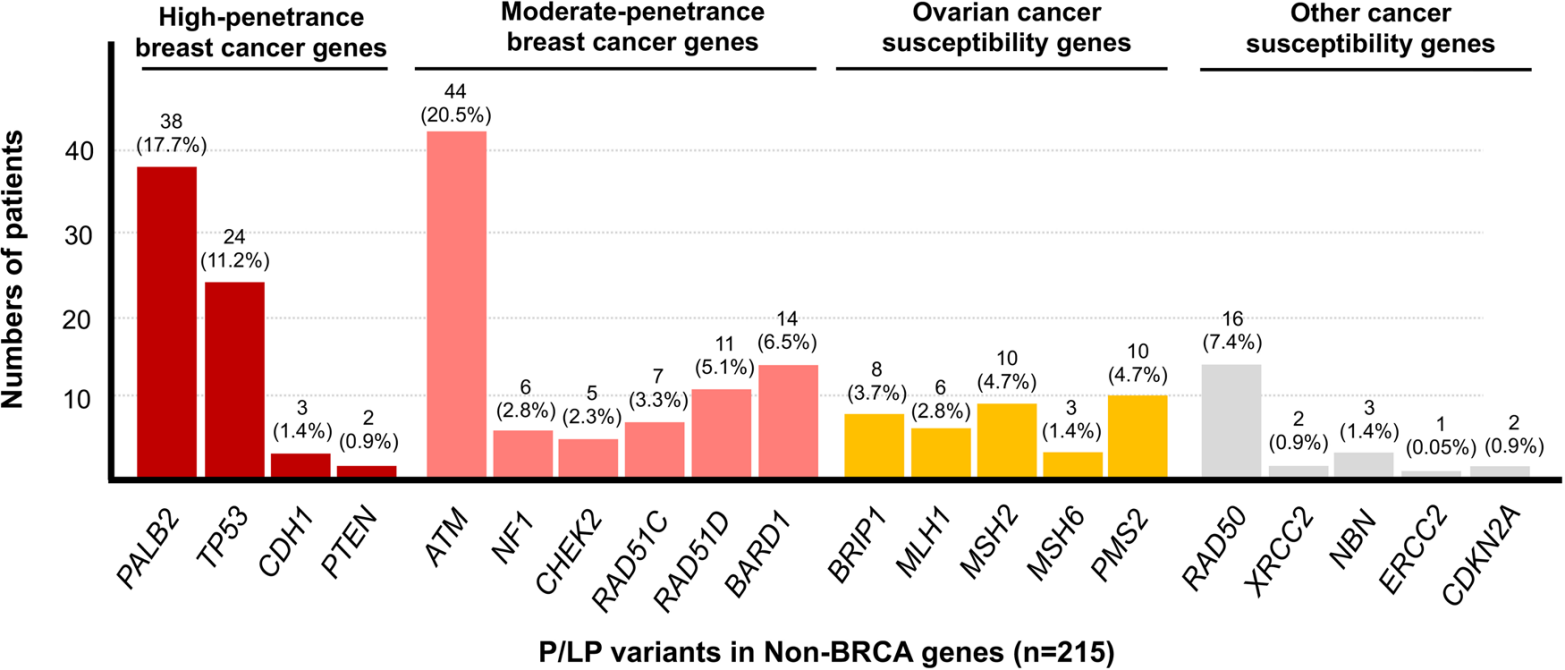
Distribution of identified non-*BRCA* P/LP variants in 214 HBOC patients.

From 214 cancer patients with non-*BRCA* P/LP variants, 215 P/LP variants were identified (Fig. 2). *ATM* was the most commonly identified gene with P/LP variants in 44 individuals (20.5%), followed by *PALB2* (*n* = 38) and *TP53* (*n* = 24). Other mutated genes are categorized in Fig. 2. A summary of patients’ and family history according to each *BRCA* and non-*BRCA* variant is demonstrated in Table 2.

**Table 2:** List of pathogenic/likely pathogenic gene(s), variants, classification, and patient’s history

Gene (Ref.)	Gene alteration	Protein alteration	Variant classification	Cancer spectrum in Patients	Family history of cancer
*BRCA1* (NM_007294.3) Median age at diagnosis 46 years, IQR 17 years	c.1 A > G	p.Met1Val	Pathogenic	Breast	Breast, bile duct
	c.53 T > C	p.Met18Thr	Likely pathogenic	Ovary	-
	c.68_69delAG	p.Glu23Valfs*17	Pathogenic	Breast	-
	c.83_84delTG	p.Leu28Argfs*	Pathogenic	Breast, ovary	-
	c.142delC	p.His476Metfs*2	Pathogenic	Breast	-
	c.212 G > T	p.Arg71Met	Pathogenic	Breast	Breast
	c.241 C > T	p.Gln81*	Pathogenic	Breast	-
	c.440delT	p.Leu147Cysfs*16	Pathogenic	Breast	Breast, ovary
	c.500_503delCAAA	p.Thr167Serfs*66	Pathogenic	Ovary	Ovary, bone (sarcoma)
	c.772delG	p.Asp258Thrfs*29	Pathogenic	Breast	-
	c.1053delA	p.Glu352Asnfs*22	Likely pathogenic	Ovary	-
	c.1155 G > A	p.Trp385*	Pathogenic	Breast	Breast, ovary
	c.1265_1266dupAT	p.Ser423Ilefs*8	Pathogenic	Ovary, breast	Lung, ovary
	c.1426delC	p.His476Metfs*2	Pathogenic	Breast	Breast
	c.1504_1508delTTAAA	p.Leu502Alafs*2	Pathogenic	Breast	Breast, ovary
	c.1542_1550delinsCG	p.Glu515Valfs*15	Pathogenic	Breast, ovary	-
	c.1550delinsCG	p.Glu515Valfs*15	Pathogenic	Ovary	Ovary
	c.1663_1664insTC	p.Arg388Serfs*7	Pathogenic	Breast	-
	c.1889delA	p.Asn630Ilefs*2	Pathogenic	Breast	Ovary, breast
	c.2101_2102delAA	p.Lys701Valfs*10	Pathogenic	Breast, ovary, fallopian tube	Endometrium
	c.2130delinsAA	p.Cys712Valfs*6	Pathogenic	Breast	Breast
	c.2269delG	p.Val757Phefs*8	Pathogenic	Breast	-
	c.2273 T > A	p.Leu758*	Pathogenic	Breast	-
	c.2411_2412delAG	p.Gln804Leufs*5	Pathogenic	Breast	-
	c.2500 G > T	p.Gly834*	Pathogenic	Ovary	-
	c.2635 G > T	p.Glu879*	Pathogenic	Breast	Breast
	c.2643dupA	p.Cys882Metfs*2	Pathogenic	Breast	-
	c.2766delA	p.Val923Leufs*77	Pathogenic	Breast	-
	c.2896dupA	p.lle966Asnfs*5	Likely pathogenic	Ovary, fallopian tube, endometrium	Ovary, prostate
	c.3020 C > A	p.Ser1007*	Pathogenic	Breast	-
	c.3037 G > T	p.Glu1013*	Pathogenic	Breast, ovary	-
	c.3049 G > T	p.Glu1017*	Pathogenic	Prostate, breast	Prostate, stomach, pancreas, thyroid, breast, ovary, colon
	c.3139_3140dupGT	p.Gly1048*	Pathogenic	Breast, pancreas	Lung
	c.3179delA	p.Ile1061*	Pathogenic	Breast	Breast
	c.3181delA	p.Ile1061*	Pathogenic	Breast, ovary	Bladder
	c.3214delC	p.Leu1072*	Pathogenic	Breast	Pancreas, ovary
	c.3403 C > T	p.Gln1135*	Pathogenic	Breast, thyroid, ovary	Ovary
	c.3424delG	p.Ala1142Hisfs*13	Pathogenic	Breast, ovary	-
	c.3598 C > T	p.Gln1200*	Pathogenic	Breast	-
	c.3637_3638delGA	p.Glu1213Argfs*3	Pathogenic	Breast	-
	c.3647 T > A	p.Leu1216*	Pathogenic	Breast	Breast
	c.3661 G > T	p.Glu1221*	Pathogenic	Breast	Pancreas, Ovary
	c.3694_3695delinsC	p.Gly1232Leufs*3	Pathogenic	Breast	Prostate
	c.3748 G > T	p.Glu1250*	Pathogenic	Breast, ovary	Colon, breast, ovary, cervix
	c.3755delT	p.Leu1252Argfs*12	Likely pathogenic	Breast, rectum	-
	c.3756_3759delGTCT	p.Ser1253Argfs*	Pathogenic	Breast, ovary	Breast, colon
	c.3770_3771delAG	p.Glu1257Glyfs*9	Pathogenic	Ovary	Ovary
	c.3882_3885delCTTG	p.Leu1295Phefs*11	Pathogenic	Breast, ovary	Breast
	c.4065_4068delTCAA	p.Asn1355Lysfs	Pathogenic	Breast, endometrium, sarcoma	
	c.4327 C > T	p.Arg1443*	Pathogenic	Breast	Peritoneum
	c.4392delT	p.Ile1465*	Pathogenic	Breast	-
	c.4506_4507insC	p.Ser1503Leufs*4	Likely pathogenic	Ovary	-
	c.4523 G > A	p.Trp1508*	Pathogenic	Breast, ovary	-
	c.4547 G > A	p.Arg1516Lys	Pathogenic	Breast	Breast, colon, lung
	c.4689 C > G	p.Tyr1563*	Pathogenic	Breast, ovary	Breast, ovary
	c.5030_5033delCTAA	p.Thr1677Ilefs*2	Pathogenic	Breast, pancreas	-
	c.5053 A > G	p.Thr1685Ala	Pathogenic	Breast	-
	c.5072 C > A	p.Thr1691Lys	Pathogenic	Ovary	Breast, ovary
	c.5114 T > C	p.Leu1705Pro	Pathogenic	Breast	-
	c.5251 C > T	p.Arg1751*	Pathogenic	Breast	-
	c.5362 G > T	p.Gly1788Cys	Pathogenic	Breast	-
	c.5406delA	p.Gly1803Valfs*31	Likely pathogenic	Breast	-
	c.5511 G > T	p.Trp1837Cys	Likely pathogenic	Breast, ovary	-
	c.5512delG	p.Val1838fs	Pathogenic	Breast	-
	c.5574 G > T	p.Trp1858Cys	Likely pathogenic	Breast	Breast, ovary
	c.8494 C > T	p.Arg2832Cys	Pathogenic	Breast	-
	c.8915delT	p.Leu2972Cysfs*4	Pathogenic	Breast	Breast
	c.135-1 G > C	Splice site variant	Likely pathogenic	Breast	-
	c.212+1 G > A	Splice site variant	Pathogenic	Breast, ovary	Breast, ovary
	c.213-12 A > G	Splice site variant	Likely pathogenic	Breast	Breast
	c.4357+1 G > A	Splice site variant	Pathogenic	Breast	Breast
	c.4357+6 T > C	Splice site variant	Pathogenic	Ovary	-
	c.4485-1 G > A	Splice site variant	Pathogenic	Ovary, breast	-
	c.4676-2 A > G	Splice site variant	Pathogenic	Ovary	
	c.4986+1 G > T	Splice site variant	Pathogenic	Ovary	Breast, ovary
	c.5074+3 A > G	Splice site variant	Likely pathogenic	Breast, ovary	Ovary
	Exon 2–12 deletion	Copy number variation		Breast	-
	Exon 5–7 duplication	Copy number variation		Pancreas	-
	Exon 16-18 deletion	Copy number variation		Breast	Breast
	Exon 20-21 deletion	Copy number variation		Breast	-
	Exon 21-22 deletion	Copy number variation		Breast, ovary	-
	Exon 23 deletion	Copy number variation		Breast	-
*BRCA2* (NM_000059.3) Median age at diagnosis 46 years, IQR 20 years	c.18_19delAG	p.Arg8Alafs*5	Pathogenic	Breast	-
	c.22_23delAG	p.Arg8Alafs*5	Pathogenic	Breast, ovary, pancreas	Breast
	c.151 G > T	p.Glu51*	Pathogenic	Breast	-
	c.157 A > T	p.Lys53*	Pathogenic	Breast	Breast
	c.346delA	p.Ser116Valfs*5	Pathogenic	Breast	Breast, ovary, prostate
	c.451_454dupGTAA	p.Thr152fs	Likely pathogenic	Breast (male), breast	-
	c.755_758delACAG	p.Asp252Valfs*24	Pathogenic		
	c.1270_1286del	p.Ser424Argfs*22	Pathogenic	Breast	-
	c.1324_1325insAG	p.Ser442Ter	Likely pathogenic	Breast	-
	c.1399_1402delAAGA	p.Lys467Glufs*17	Pathogenic	Breast	Breast
	c.1405_1406delGA	p.Asp469*	Pathogenic	Breast	-
	c.1763_1766delATAA	p.Asn588Serfs*	Pathogenic	Breast	-
	c.1773_1776delTTAT	p.lle591Metfs*	Pathogenic	Breast, prostate	-
	c.1813delA	p.Ile605Tyrfs*9	Pathogenic	Breast	
	c.2327delA	p.Lys776Argfs*7	Pathogenic	Breast, ovary	-
	c.2372 C > G	p.Ser791*	Pathogenic	Breast	-
	c.2781_2784delGGTT	p.Met927llefs*32	Likely pathogenic	Breast	Lung
	c.2808_2811delACAA	p.Ala938Profs*21	Pathogenic	Breast	Breast (male), ovary
	c.2925delA	p.Glu866Lysfs*8	Pathogenic	Breast	-
	c.2990 T > G	p.Leu997*	Pathogenic	Breast	Breast
	c.3109 C > T	p.Gln1037*	Pathogenic	Breast	Breast, oral cavity
	c.3449delC	p.Thr1150llefs*	Likely pathogenic	Breast	Breast
	c.3716_3717delAA	p.Lys1239Thrfs*3	Pathogenic	Breast, ovary, colon	Breast, prostate, lung, leukemia
	c.3847_3848delGT	p.Val1283Lysfs*2	Pathogenic	Breast, ovary	Peritoneum
	c.3865_3868delAAAT	p.Lys1289Alafs*3	Pathogenic	Breast	Breast, endometrium
	c.4115_4116insC	p.Met1373Tyrfs*9	Likely pathogenic	Breast	Breast
	c.4126 G > T	p.Gly1376*	Pathogenic	Breast	-
	c.4245delG	p.Glu1415Aspfs*4	Pathogenic	Breast	-
	c.4936_4939delGAAA	p.Glu1546Glnfs*23	Pathogenic	Breast	Breast
	c.5087_5090dupGAAT	p.lle1697Metfs*4	Pathogenic	Breast	-
	c.5116_5119delAATA	p.Asn1706Leufs*5	Pathogenic	Breast, pancreas	Breast, colon
	c.5130_5133delTGTA	p.Tyr1710*	Pathogenic	Breast	-
	c.5645 C > A	p.Ser1882*	Pathogenic	Breast, thyroid, colon	Breast, prostate
	c.5771_5774delTTCA	p.Ile1924Argfs*38		Breast	Breast, liver, colon
	c.5980 C > T	p.Gln1994*	Pathogenic	Breast	-
	c.6154delT	p.Ser2052Hisfs*18	Pathogenic	Breast	-
	c.6266delA	p.Glu2089Glyfs*30	Likely pathogenic	Breast	-
	c.6298_6299insA	p.Asn2101Lysfs*10	Pathogenic	Breast	Breast, pancreas, endometrium
	c.6405_6409delCTTAA	p.Asn2135Lysfs*3	Pathogenic	Breast	-
	c.6486_6489delACAA	p.Lys2162Asnfs*5	Pathogenic	Breast, ovary	Breast
	c.6532dupC	p.His2178Profs*11	Pathogenic	Breast	Breast, prostate, colon
	c.6541 G > T	p.Gly2181*	Pathogenic	Breast	-
	c.6673delA	p.Thr2225Glnfs*4	Pathogenic	Breast	Breast
	c.6777_6778delTG	p.Asn2259Lysfs*33	Pathogenic	Breast, ovary	Colon, endometrium
	c.6896delA	p.Asn2299Ilefs*6	Pathogenic	Breast	-
	c.6952 C > T	p.Arg2318*	Pathogenic	Breast, ovary	-
	c.6997_6998delGT	p.Val2333Thrfs*6	Pathogenic	Breast	Ovary, lung, neuroendocrine
	c.7185_7188delCTTG	p.His2395Glnfs*71	Pathogenic	Breast	Breast
	c.7185_7190delinsAG	p.His2395Glnfs*71	Pathogenic	Breast	Breast, brain
	c.7288 G > T	p.Glu2430*	Pathogenic	Breast	Breast, leukemia, peritoneum
	c.7544_7545insA	p.Ser2516Ilefs*23	Pathogenic	Breast	-
	c.7558 C > T	p.Arg2520*	Pathogenic	Pancreas, breast, lung	-
	c.7643_7644delAT	p.His2548Leufs*5	Pathogenic	Breast	-
	c.7673_7674delAG	p.Glu2558Valfs*7	Pathogenic	Breast	Bladder, colon
	c.7767delC	p.Ser2590Profs*58	Pathogenic	Breast, endometrium	Breast, thyroid
	c.7999delA	p.Ser2667Alafs*6	Pathogenic	Breast	Breast, liver
	c.8023delA	p.Ile2675*	Pathogenic	Breast	Breast
	c.8168 A > C	p.Asp2723Ala	Pathogenic	Breast	-
	c.8191 C > T	p.Gln2731*	Pathogenic	Breast	-
	c.8837_8841delTGGAA	p.Leu2946Tyrfs*2	Pathogenic	Prostate, breast (male), esophagus	-
	c.8854_8855insT	p.Met2952Ilefs*5	Pathogenic	Breast	Breast, ovary, colon
	c.8890dupA	p.Arg2964Lysfs*54	Pathogenic	Breast, ovary	-
	c.8915delT	p.Leu2972Cysfs*4	Pathogenic	Breast	Breast
	c.9097delA	p.Thr3033Leufs*29	Pathogenic	Breast	Breast
	c.9154 C > T	p.Arg3052Trp	Pathogenic	Breast	Breast, ovary
	c.9382 C > T	p.Arg3128*	Pathogenic	Breast	Breast, colon
	c.7007+1 G > C	Splice site variant	Pathogenic	Breast	-
	c.7008-1 G > A	Splice site variant	Pathogenic	Breast	-
	c.7618-1 G > A	Splice site variant	Pathogenic	Breast, colon	-
	c.8331+2 T > A	Splice site variant	Pathogenic	Ovary, fallopian tube	-
	c.8953+1 G > C	Splice site variant	Likely pathogenic	Breast, ovary	Breast
	Exon 5–7 deletion	Copy number variation		Breast	-
	Exon 19-20 deletion	Copy number variation		Breast	Lung, Breast
	Exon 25-27 deletion	Copy number variation		Breast	Breast
	Exon 26-27 deletion	Copy number variation		Breast	Breast
*ATM* (NM_000051.3) Median age at diagnosis 47 years, IQR 14 years	c.1 A > G	p.Met1Val	Pathogenic	Breast	-
	c.769 G > T	p.Glu257*	Pathogenic	Breast	-
	c.875 C > T	p.Pro292Leu	Likely pathogenic	Breast, ovary	-
	c.1339 C > T	p.Arg447*	Pathogenic	Breast, pancreas, endometrium	Pancreas
	c.1402_1403delAA	p.Lys468Glufs*18	Pathogenic	Lung, ovary, pancreas	Colon
	c.1655delC	p.Pro552Glnfs*4	Pathogenic	Breast	-
	c.2086 G > T	p.Gly696*	Likely pathogenic	Sarcoma, breast	Breast, ovary, lung
	c.2341 C > T	p.Gln781*	Pathogenic	Breast	-
	c.2413 C > T	p.Arg805*	Pathogenic	Breast	-
	c.3693_3697delATCTT	p.Leu1231Phefs*13	Likely pathogenic	Ovary	Prostate
	c.3712_3716delTTATT	p.Leu1238fs	Likely pathogenic	Breast	Breast
	c.4335dupT	p.Val1446Cysfs*2	Pathogenic	Pancreas	-
	c.4664delT	p.Leu1555Pro*	Pathogenic	Breast	-
	c.4852 C > T	p.Arg1618*	Pathogenic	Breast	Breast
	c.5040dupT	p.Ile1681Tyrfs*11	Pathogenic	Pancreas, colon, breast	Pancreas
	c.5692 C > T	p.Arg1898*	Pathogenic	Breast	Breast
	c.7542 T > G	p.Tyr2514*	Likely pathogenic	Breast	Stomach, colon
	c.7843 C > T	p.Gln2615*	Likely pathogenic	Breast	Colon
	c.7886_7890delTATTA	p.Ile2629Serfs*25	Pathogenic	Breast	-
	c.8287 C > T	p.Arg2763*	Pathogenic	Breast	-
	c.8395_8404delTTTCAGTGCC	p.Phe2799Lysfs*4	Pathogenic	Breast	-
	c.8435_8436delCT	p.Ser2812Phefs*2	Likely pathogenic	Pancreas, colon	-
	c.8494 C > T	p.Arg2832Cys	Pathogenic	Breast	-
	c.8824_8834delCAGGAAACTCT	p.Gln2942Valfs*10	Pathogenic	Breast, lung	Lung
	c.8879 G > A	p.Trp2960*	Pathogenic	Breast, colon	CNS (brain)
	c.2377-2 A > G	Splice site variant	Pathogenic	Breast	-
	c.6453-2 A > G	Splice site variant	Likely pathogenic	Breast, colon	-
	c.3994-1 G > T	Splice site variant	Pathogenic	Breast	Breast
	Exon 62_63 deletion	Copy number variation		Breast (male), colon	-
*TP53* (NM_000546.5) Median age at diagnosis 33 years, IQR 15.5 years	c.325 T > G	p.Phe109Val	Likely pathogenic	Breast	-
	c.371dupG	p.Cys124Trpfs*25	Pathogenic	Breast	Lung, HCC
	c.374 C > T	p.Thr125Met	Pathogenic	Breast, thyroid, sarcoma, urothelial, pancreas	Lung, brain, colon, kidney, thyroid (papillary)
	c.375 G > A	p.Thr125=	Pathogenic	Breast	-
	c.422 G > T	p.Cys141Phe	Pathogenic	Breast	Breast
	c.524 G > A	p.Arg175His	Pathogenic	Breast	-
	c.528 C > A	p.Cys176*	Pathogenic	-	Breast, lung, CNS (brain)
	c.538 G > A	p.Glu180Lys	Likely pathogenic	Breast	Lung
	c.586 C > T	p.Arg196*	Pathogenic	Breast	Esophagus, cervix
	c.637 C > T	p.Arg213*	Pathogenic	Breast	Breast
	c.659 A > G	p.Tyr220Cys	Pathogenic	Breast, endometrium	-
	c.733 G > A	p.Gly245Ser	Pathogenic	Breast, adrenocortical	Breast, head/neck cancer
	c.742 C > T	p.Arg248Trp	Pathogenic	Breast, lymphoma, colon	Breast, leukemia, CNS (brain), esophagus, bone, lung
	c.799 C > T	p.Arg267Trp	Pathogenic	Breast	-
	c.818 G > A	p.Arg273His	Pathogenic	Breast, leukemia	Pancreas, kidney
	c.832 C > T	p.Pro278Ser	Likely pathogenic	Breast	-
	c.839 G > T	p.Arg280Ile	Likely pathogenic	Breast	-
	c.1024 C > T	p.Arg342*	Pathogenic	Breast, colon	-
	c. 96 + 1 G > A	Splice site variant	Pathogenic	Breast, CNS (brain)	-
	c.559+1 G > A	Splice site variant	Pathogenic	Breast	-
	Exon 10_11 deletion	Copy number variation		Breast	Breast
*PALB2* (NM_024675.3) Median age at diagnosis 49.5 years, IQR 12.8 years	c.7 G > T	p.Glu3*	Pathogenic	Breast	-
	c.276_279dupTGGA	p.Glu94Trpfs*9	Likely pathogenic	Breast	Breast
	c.626delC	p.Ser209Phefs*14	Likely pathogenic	Breast	-
	c.758dupT	p.Ser254Ilefs*3	Pathogenic	Breast	Leukemia
	c.778 C > T	p.Gln260*	Pathogenic	Breast	-
	c.1059delA	p.Lys353Asnfs*3	Pathogenic	Breast	-
	c.1660G>T	p.Glu554*	Likely pathogenic	Breast	-
	c.2255_2267dupGACGA ACTTGCTG	p.Cys756Trpfs*21	Pathogenic	Breast	Breast
	c.2257 C > T	p.Arg753Ter	Pathogenic	Breast	Prostate
	c.2411_2412delCT	p.Ser804Cysfs*10	Pathogenic	Breast	-
	c.2503delT	p.Ser835Profs*16	Likely pathogenic	Breast	-
	c.2520_2521delAA	p.Glu840Aspfs*8	Pathogenic	Breast	-
	c.2546delG	p.Arg753*	Pathogenic	Breast	-
	c.2704_2707dupGATG	p.Ala903Glyfs*26	Likely pathogenic	Breast	-
	c.2968 G > T	p.Glu990*	Pathogenic	Breast	-
	c.3267_3268delGT	p.Phe1090Serfs*6	Likely pathogenic	Ovary	-
	c.3426_3429delACTT	p.Leu1142Phefs*20	Pathogenic	Breast	-
	c.3625 T > G	p.Leu1209Val	Pathogenic	Breast	-
	c.109-2 A > C	Splice site variant	Pathogenic	Breast	-
	c.1684+1 G > A	Splice site variant	Pathogenic	Breast, lung	-
	c.2515-2 A > G	Splice site variant	Likely pathogenic	Breast	-
	c.3350+5 G > A	Splice site variant	Likely pathogenic	Breast	-
	Exon 2_4 deletion	Copy number variation		Breast	-
	Exon 8 deletion	Copy number variation		Breast	Breast
	Exon 1_10 deletion	Copy number variation		Breast	-
*RAD50* (NM_005732.4) Median age at diagnosis 47 years, IQR 12.5 years	c.1111delA	p.Ile371Phefs*8	Pathogenic	Breast	Breast
	c.1751C>G	p.Ser584*	Likely pathogenic	Breast, stomach	-
	c.2165delA	p.Lys722Argfs*14	Pathogenic	Breast	Cervix
	c.2263 C > T	p.Gln755*	Likely pathogenic	Breast	-
	c.2983_2986delGAAA	p.Glu995Argfs*2	Pathogenic	Breast	Breast
	c.3528delT	p.Asp1177Ilefs*11	Likely pathogenic	Breast	Lung
	c.3553 C > T	p.Arg1185*	Pathogenic	Breast	-
	c.3598 C > T	p.Arg1200*	Pathogenic	Breast	Breast, liver
	c.3715 C > T	p.Arg1239*	Likely pathogenic	Breast	-
	c.7768 C > T	p.Lys50Asnfs*29	Likely pathogenic	Breast	-
	Exon 2 deletion	Copy number variation		Breast	Colon
*RAD51C* (NM_058216.3) Median age at diagnosis 54.5 years, IQR 15.5 years	c.394dupA	p.Thr132Asnfs*23	Pathogenic	Breast, endometrium	Breast, lung, esophagus
	c.145+1 G > T	Splice site variant	Pathogenic	Breast	-
	c.405-1 G > A	Splice site variant	Pathogenic	Pancreas	Breast
	c.905-2 A > C	Splice site variant	Pathogenic	Breast, pancreas	Breast
*RAD51D* (NM_002878.4) Median age at diagnosis 42.5 years, IQR 12 years	c.27 C > A	p.Cys9*	Pathogenic	Breast	-
	c.270_271dupTA	p.Lys91Ilefs*13	Pathogenic	Breast, ovary	Breast (male, female), lung, Liver, colon
	c.385 C > T	p.Gln129*	Pathogenic	Ovary	Breast
	c.694 C > T	p.Arg232*	Pathogenic	Breast	-
	Exon 1_3 deletion	Copy number variation		Breast	-
	Exon 5_10 deletion	Copy number variation		Breast	-
*NF1* (NM_001042492.3) Median age at diagnosis 45 years, IQR 23 years	c.1658A>G	p.His553Arg	Pathogenic	Breast	-
	c.2735_2750del	p.Gln912Leufs*7	Likely pathogenic	Breast	-
	c.4375 G > C	p.Glu1459Gln	Pathogenic	Breast	-
	c.4600 C > T	p.Arg1534Ter	Pathogenic	Breast	-
	c.1062+1 G > T	Splice site variant	Pathogenic	Breast	-
	c.1846-1 G > T	Splice site variant	Likely pathogenic	Breast	-
*PTEN* (NM_000314.8) Median age at diagnosis 43.5 years IQR 0.75 years	c.697 C > T	p.Arg233*	Pathogenic	Breast	-
	c.842_843insCTG	p.Pro281_Gly282insTer	Likely pathogenic	Breast	Breast, liver
*CDH1* (NM_004360.5) Median age at diagnosis 57 years IQR 12.5 years	c.1137 G > A	p.Thr379=	Likely pathogenic	Breast, colon	Breast, lung
	c.1792C>T	p.Arg598*	Pathogenic	Breast	-
*BARD1* (NM_000465.4) Median age at diagnosis 45.5 years IQR 10.5 years	c.69_70delins(25)	p.Ala25Glyfs*41	Pathogenic	Breast	Liver, thyroid, leukemia
	c.76delA	p.Met26Trpfs*32	Likely pathogenic	Breast	-
	c.334 C > T	p.Arg112*	Pathogenic	Breast	-
	c.593delC	p.Ala198Valfs*14	Likely pathogenic	Breast	-
	c.808 G > T	p.Glu270*	Pathogenic	Breast, colorectum	Endometrium
	c.1348_1349delinsCAT	p.Asn450Hisfs*4	Pathogenic	Breast, pancreas	-
	c.1811-1 G > A	Splice site variant	Likely pathogenic	Breast	Breast, lung
*BRIP1* (NM_032043.3) Median age at diagnosis 40.8 years IQR 20.5 years	c.644_645delCT	p.Ser215*	Pathogenic	Breast	-
	c.1066 C > T	p.Arg356*	Pathogenic	Breast	Breast
	c.1315 C > T	p.Arg439*	Pathogenic	Breast	Liver, brain
	c.1343 G > A	p.Trp448*	Pathogenic	Breast	-
	c.2431_2432dupCT	p.Pro812Tyrfs*15	Likely pathogenic	Breast	-
	c.3072delG	p.Ser1025His	Likely pathogenic	Breast	-
	c.2097+1dupG	Splice site variant	Likely pathogenic	Breast	-
*CHEK2* (NM_007194.4) Median age at diagnosis 43 years IQR 3.3 years *CDKN2A* (NM_000077.5) Median age at diagnosis 68.5 years IQR 3.5 years	c.1238 T > G	p.Leu413*	Pathogenic	Breast	-
	c.1008+2 T > A	Splice site variant	Likely pathogenic	Breast	-
	c.1096-2 A > T	Splice site variant	Likely pathogenic	Breast	-
	Exon 9 deletion	Copy number variation		Breast	Breast
	c.44 G > A	p.Trp15*	Pathogenic	Breast	Breast
	c.367delC	p.His123Ilefs*23	Likely pathogenic	Pancreas	-
*MLH1* (NM_000249.4) Median age at diagnosis 49 years IQR 16.5 years *MSH2* (NM_000251.3) Median age at diagnosis 50 years IQR 6.8 years	c.2011 G > T	p.Glu671*	Pathogenic	Ovary	Colon
	c.790+1G > A	Splice site variant	Pathogenic	Ovary, endometrium, colon, prostate, bladder	Breast, endometrium,
	c.884+4 A > G	Splice site variant	Pathogenic	Breast	-
	Exon 7-8 deletion	Copy number variation		Breast, ovary	Colon, cervix
	Exon 15 deletion	Copy number variation		Breast	-
	c.811_814delTCTG	p.Ser271Argfs*2	Pathogenic	Breast	-
	c.1237 C > T	p.Gln413*	Pathogenic	Ovary	Breast, endometrium
	c.1786_1788delAAT	p.Asn596del	Pathogenic	Breast, colorectal	Breast, colorectal, cervix
	c.1930delG	p.Val644Phefs*41	Pathogenic	Breast, endometrium	Ovary, bladder
	c.2018delG	p.Gly673Glufs*12	Likely pathogenic	Ovary, colon	-
	c.2432 T > G	p.Leu811*	Pathogenic	Breast	-
	c.2554 G > T	p.Glu852*	Likely pathogenic	Ovary	-
	c.942+3 A > T	Splice site variant	Likely pathogenic	Breast, ovary, endometrium	Breast, colorectal
	c.1661+1 G > A	Splice site variant	Likely pathogenic	Ovary	Colon
	Exon 7-8 deletion	Copy number variation		Breast, colon	Endometrium, colon
*MSH6* (NM_000179.3) Median age at diagnosis 63 years IQR 13 years	c.3261dupC	p.Phe1088fs	Pathogenic	Breast	-
	c.1145delA	p.His382Profs*29	Pathogenic	Prostate, colon	Endometrium, breast, colon
	c.628-1 G > A	Splice site variant	Likely pathogenic	Breast (male)	-
*PMS2* (NM_000535.7) Median age at diagnosis 48 years IQR 15.3 years	c.1 A > G	p.Met1Val	Pathogenic	Ovary	-
	c.325dupG	p.Glu109Glyfs*30	Pathogenic	Ovary	Colon
	c.451delC	p.Arg151Alafs*50	Pathogenic	Breast	-
	c.943 C > T	p.Arg315*	Pathogenic	Ovary	-
	c.1864_1865del	p.Met622Glufs*5	Pathogenic	Breast	Lung, pancreatic
	c.2243-2246delAGAA	p.Lys748Metfs*19	Pathogenic	Breast	-
	c.2444 C > T	p.Ser815Leu	Likely pathogenic	Breast	-
	c.706-1 G > T	Splice site variant	Pathogenic	Breast	Breast
	c.2174+1 G > A	Splice site variant	Pathogenic	Breast	Breast
*NBN* (NM_002485.5) Median age at diagnosis 54 years IQR 11 years	c.5 G > A	p.Trp2*	Pathogenic	Breast	-
	c.89delA	p.Asn30Thrfs*5	Likely pathogenic	Breast	-
	c.1516 C > T	p.Gln506*	Pathogenic	Breast	-
*ERCC2* (NM_000400.4)	c.2164 C > T	p.Arg722Trp	Pathogenic	Ovary	Ovary
*XRCC2* (NM_005431.2)	c.1 A > T	p.Met1Leu	Likely pathogenic	Breast	-
	c.323_326delTCAA	p.108Asnfs*25	Likely pathogenic	Breast	-

### Distribution of cancers in patients with P/LP variants

Of 537 breast cancer patients with P/LP variants, P/LP variants in moderate-to-high penetrance breast cancer susceptibility genes were identified in 491 individuals. Meanwhile, there were 78 ovarian, 14 pancreatic, and six prostate cancer patients with P/LP variants. The detection rate of P/LP variants was 19.8% in ovarian cancer patients, followed by pancreatic (14%), breast (11.8%), and prostate cancer (7.1%).

Fifty-four patients had multiple primary cancers, consisting of 27 patients with HBOC-related cancers. The other 27 individuals had additional cancers not in the HBOC spectrum, including colorectal cancer (*n* = 13), endometrial cancer (*n* = 11), sarcomas (*n* = 4) (see Supplementary Table 1). In addition, 162 patients (26.6%) had a history of cancer in their families.

### Multi-gene panel testing in breast cancer and NCCN indication fulfillment

Of 491 breast cancer patients with P/LP variants in breast cancer susceptibility genes, 72.3% (*n* = 355) were *BRCA1* or *BRCA2* variants, while 139 patients had non-*BRCA* variants. *ATM* was the most commonly identified non-*BRCA* gene (*n* = 37, 26.6%), followed by *PALB2* (*n* = 36, 25.9%). Details of P/LP variants in other non-BRCA genes are shown in Table 1.

A total of 432 breast cancer patients with P/LP variants (80.4%) fulfilled the 2023 NCCN testing criteria for high-penetrance breast cancer susceptibility genes (Table 3). The most common indication was early-onset breast cancer (*n* = 365), followed by family history of HBOC-related cancers (*n* = 102), multiple primary breast cancer (*n* = 57), triple-negative breast cancer (*n* = 50), primary breast and ovarian cancers (*n* = 22), and male breast cancer (*n* = 10). The proportion of patients with P/LP variants in *BRCA* and non-*BRCA* genes who met each criterion is shown in Table 3.

**Table 3:** Breast cancer patients with P/LP variants categorized by NCCN eligibility criteria for germline high-penetrance breast cancer susceptibility genes testing, and indications for genetic testing in breast cancer patients (number of all patients is less than a combination of each group due to the presence of double mutations)

	All patients with P/LP variants (*n* = 537)	Patients with P/LP *BRCA1/2* variants (*n* = 355)	Patients with P/LP non-*BRCA* variants (*n* = 187)
Compatible with 2023 NCCN genetic testing criteria	432 (80.4%)	296 (83.4%)	140 (74.9%)
Indications for genetic testing in breast cancer patients			
• Early onset breast cancer (age ≤50 years)	365/432 (84.5%)	241/296 (81.4%)	127/140 (90.7%)
• Strong family history of HBOC-related cancer	102/432 (23.6%)	84/296 (28.4%)	19/140 (13.6%)
• Multiple primary (synchronous or metachronous) breast cancer	50/432 (11.6%)	42/296 (14.2%)	10/140 (7.1%)
• Triple-negative breast cancer	57/432 (13.2%)	40/296 (13.5%)	18/140 (12.9%)
• Male breast cancer	10/432 (2.3%)	9/296 (3.0%)	2/140 (1.4%)
• Breast and ovarian cancer	22/432 (5.1%)	17/296 (5.7%)	5/140 (3.6%)

In this cohort, 908 breast cancer patients who received genetic testing did not meet current NCCN criteria. In this group, 105 patients (11.6%) harbored P/LP variants in *BRCA1/2* (*n* = 59) and non-*BRCA* genes (*n* = 47). Thirty-two patients with P/LP variants were found in moderate-to-high penetrance non-*BRCA* genes. Clinical data on patients not meeting the criteria are available in Supplementary Table 2.

### Multi-gene panel testing in other HBOC-related cancers

Of 78 ovarian cancer patients with germline P/LP variants, 40 had *BRCA1*, 17 had *BRCA2*, and 22 had non-*BRCA* variants (Table 1). P/LP variants in mismatch repair (MMR) genes (*MLH1, MSH2, and PMS2*) accounted for 14.4% (*n* = 11) (see Supplementary Table 3).

There were 14 pancreatic cancer patients with germline P/LP variants, including 6 with *BRCA1/2* variants and 8 with non-*BRCA* variants. Six out of 84 prostate cancer patients also had P/LP variants. Detailed results are shown in Table 1.

### Detection of double pathogenic variants in HBOC patients

This cohort identified ten individuals with double pathogenic variants (Table 4). Nine patients had concomitant non-*BRCA* and *BRCA1* or *BRCA2* P/LP variants. One patient found both pathogenic *RAD51C* and *CDKN2A* variants.

**Table 4:** Identified double P/LP variants in HBOC-related cancer patients

Variants 1	Classification	Variants 2	Classification	Cancer in patients
*BRCA1*: c.3847_3848 delGT	Pathogenic	*BRCA2*: c.772delG	Pathogenic	Breast
*BRCA1*: c.213-12 A > G	Pathogenic	*RAD50*: c.2980_2983 delAAAG	Pathogenic	Breast
*BRCA1*: c.83_84delTG	Pathogenic	*RAD51D*: c.385 C > T	Pathogenic	Ovary
*BRCA1*: c.4986+1 G > T	Pathogenic	*ERCC2*: c.2164 C > T	Pathogenic	Ovary
*BRCA2:* CNV exon 25-27 deletion	Likely pathogenic	*ATM*: c.5692 C > T	Pathogenic	Breast
*BRCA2:* c.1405_1406 delGA	Pathogenic	*ATM*: c.7886_7890 delTATTA	Pathogenic	Breast
*BRCA2*: c.631+3 A > G	Likely pathogenic	*ATM*: c.2086 G > T	Pathogenic	Breast (bilateral)
*BRCA2*: c.7767delC	Pathogenic	*RAD50*: c.3715 C > T	Pathogenic	Breast
*BRCA2:* c.451_454dupGTAA	Likely pathogenic	*MSH6:* c.628-1 G > A	Pathogenic	Male breast
*RAD51C*: c.405-1 G > A	Pathogenic	*CDKN2A*: c.367delC	Pathogenic	Pancreas

## Discussion

This study provides insights into germline mutations and cancer phenotypes in Thai patients with HBOC spectrum and demonstrates the favorable diagnostic yield of multi-gene panel testing in Thai cancer patients. In this study, 13.4% of Thai patients with HBOC-related cancers were associated with cancer susceptibility genes. Among P/LP variants, non-*BRCA* genes accounted for 35% of these cases. The proportion of non-*BRCA* variants in breast cancer patients with germline mutation in this study is consistent with previous studies, ranging from 6.8% to 40% in high-risk breast cancer patients^11–15,19,20^. P/LP variants in non-*BRCA* genes are identified in 28.3% of breast and 28.2% of ovarian cancer patients with germline mutations. This suggests that the incorporation of additional cancer susceptibility genes in the test for Thai patients with HBOC spectrum may enhance the diagnostic yield by as much as 28% in comparison to *BRCA*-only testing. Our result aligned with a prior study that supports multi-gene panel testing among breast cancer patients^21^. Our findings may guide physicians to consider multi-gene panel testing for patients with HBOC-related cancers.

While *PALB2* and *TP53* were the most reported non-*BRCA* genes in breast cancer patients in various studies^11–14^, *ATM* was the most common non-*BRCA* gene mutations in our cohort. From previous studies, the prevalence of *ATM* P/LP variants among non-*BRCA* mutated breast cancer patients varied widely^11,13,14^. A meta-analysis also suggested a pooled prevalence of 7% in P/LP *ATM* variants among high-risk breast cancer cohorts^22^. This data may indicate a higher frequency of *ATM* carriers in the Thai population. *ATM* mutations are associated with breast and other cancer susceptibilities^23,24^ and may confer a risk of contralateral breast cancer in patients undergoing radiotherapy^25^. Despite its relevance, this gene is not included in the NCCN guidelines. Our results support the inclusion of *ATM* into the breast cancer gene panel for the Thai population.

Our study reveals that only 80% of breast cancer patients with germline mutations met the NCCN criteria for genetic testing. The observation that one-fifth of Thai breast cancer patients with pathogenic variants would miss out on testing opportunities aligned with a previous study in which NCCN criteria missed around 30% of patients with pathogenic variants^26^. It has been suggested that lowering the age threshold for universal genetic testing could improve the detection rate in breast cancer patients^27^, which was supported by the 2019 American Society of Breast Surgeons^28^. Owing to the high acceptance of genetic testing and counseling, more detection of cases would benefit treatment, screening, and prevention for patients and family members carrying pathogenic variants^29,30^.

The diagnostic yield of germline testing in Thai ovarian cancer patients was 20%, which is comparable with previous studies^31–33^. Mismatch repair (MMR) genes accounted for 14% of P/LP variants in ovarian cancer patients, consistent with the evidence that supports an association between ovarian cancer and Lynch syndrome^5,34,35^. These findings should raise physicians’ awareness of genetic testing beyond *BRCA1/2* and encourage the inclusion of MMR genes in the panel for Thai ovarian cancer patients.

Twenty-seven patients (4.4%) who fulfilled breast-ovarian cancer testing indications had a history of other cancer types. It is well known that colorectal cancer, endometrial cancer, brain tumor, and sarcoma are associated with genetic predispositions such as MMR genes or *TP53*. Many high-penetrance genes also exhibit pleiotropic clinical manifestations of other common cancers. As multi-gene panel testing for all breast cancer patients was found to be cost-effective, it may be rational to expand testing to patients with cancers in the HBOC spectrum^36^.

Lastly, we identified double pathogenic variants in ten individuals. To date, there is limited data on double mutations in cancer patients. One study found double heterozygous variants in 1.2% of hereditary breast cancer patients^37^. However, the exact prevalence is still undetermined. It is unclear if patients with double mutations would have different cancer susceptibility and clinical severity compared to single mutation carriers. However, this information can be used for proper surveillance strategies for a broader spectrum of cancers in patients and at-risk family members.

With various screening methods for breast and ovarian cancer, as well as the availability of prophylactic surgeries in most regions of Thailand, this data will properly guide physicians for personalized surveillance and preventive strategies in patients or at-risk family members. Our findings will support the rationale of implementing multi-gene panel testing beyond *BRCA1*/*2* in Thai patients with HBOC-related cancers and provide more information on the Southeast Asian population. With cost reduction and faster turnaround time, the clinical use of multi-gene panel tests for cancer will gradually increase among many low-to-middle-income countries.

This study had some limitations. Firstly, some variants were found in a small number of patients. Therefore, the clinical data associated with those variants were limited. Secondly, some clinical data was incomplete, and the follow-up duration may not have been long enough to demonstrate susceptibility to other types of cancer. Thirdly, this cohort may be biased towards breast cancer patients and away from prostate cancer. This is explained by greater awareness about genetic factors involved in breast and ovarian cancers, leading to more genetic testing in these patients. Moreover, guidelines for genetic testing in high-risk breast/ovarian cancer patients have been widely published, while genetic testing for prostate cancer has only been recommended in recent NCCN guidelines for patients with metastatic disease, strong family history, or high-risk features (e.g., very high PSA level or high Gleason score)^5,38^. In Thailand, genetic testing for prostate cancer is not included in current national guidelines, leading to underutilization in these patients. Finally, this was a single-center study with most patients from the central region of Thailand. However, Siriraj Hospital is a major cancer referral center that provides genetic testing services to other hospitals nationwide.

## Methods

### Patient recruitment and data collection

This study was approved by the Siriraj Hospital Institutional Review Board Protocol No. 418/2562(EC2) and was conducted according to Good Clinical Practice and the Declaration of Helsinki. All participants provided written informed consent. All Thai patients with any cancers in the HBOC spectrum, including breast, ovarian, pancreatic, and prostate cancers, who had germline cancer susceptibility multi-gene panel testing at Siriraj Hospital between 2016 and 2023 were included. The data of all patients with germline P/LP variants in cancer susceptibility genes were collected. Patients with known clinical syndromes of Mendelian disorders (such as neurofibromatosis, ataxia-telangiectasia, or Peutz-Jeghers syndrome) and individuals who received targeted gene testing due to known affected family members were excluded. The data regarding the types of cancers, histopathological profiles, clinical stages of cancer, age of onset, and family history were systematically collected and reviewed with 2023 NCCN guidelines for genetic/familial high-risk assessment of breast and ovarian cancers^5^. We calculated the positive rate of P/LP *BRCA* and non-*BRCA* variants using descriptive statistics for each cancer type. Additionally, we reported the clinical spectrum of breast cancer patients with non-*BRCA* P/LP variants.

### Multi-gene panel testing and variant analysis

Peripheral blood specimens from each patient were collected to extract genomic DNA for sequencing (see Supplementary 4). The comprehensive cancer panel in this study included moderate-to-high penetrance genes for breast and ovarian cancers (Table 5)^5–7,39^. Sanger sequencing and Multiplex Ligation-dependent Probe Amplification (MLPA) were performed to validate the results in all identified single nucleotide variants (SNV) and copy number variants (CNV), respectively. The variant call format (VCF) and BAM files were transferred to VarSeq – VSClinical software (Golden Helix, USA) for analysis and classification. We interpreted and classified the variants following the 2015 ACMG-AMP standards and guidelines for the interpretation of sequence variants and 2020 ACMG-ClinGen technical standards for the interpretation and reporting of constitutional copy-number variants^40,41^. All reportable variants, including P/LP variants and VUS, were systematically verified.

**Table 5:** Lists of genes in comprehensive cancer panel

Phenotype	Genes
High-penetrance genes for breast cancer	*BRCA1, BRCA2, CDH1, STK11, PALB2, TP53, PTEN*
Moderate genes for breast cancer	*ATM, CHEK2, NF1, BARD1, RAD51C, RAD51D*
Possible breast cancer genes	*NBN, RAD50, XRCC2*
Moderate-risk ovarian cancer genes	*BRIP1, MLH1, MSH2, MSH6, PMS2, EPCAM*
High-penetrance genes for other types of cancer	*APC, AXIN2, BMPR1A, CDK4, CDKN2A, FANCC, MSH2, MUTYH, NTHL1, POLD1, POLE, SMAD4, VHL*

OMIM numbers for each gene: *BRCA1* (OMIM 113705), *BRCA2* (OMIM 600185), *CDH1* (OMIM 192090), *STK11* (OMIM 602216), *TP53* (191170), *ATM* (OMIM 607585), *BRIP1* (OMIM 605882), *CHEK2* (OMIM 604373), *NF1* (OMIM 613113), *PALB2* (OMIM 610355), *BARD1* (OMIM 601593), *NBN* (OMIM 602667), *RAD50* (OMIM 604040), *XRCC2* (OMIM 600375), *RAD51C* (OMIM 602774), *RAD51D* OMIM 602954), *MLH1* (OMIM 120436), *MSH2* (OMIM 609309), *MSH6* (OMIM 600678), *PMS2* (OMIM 600259), *EPCAM* (OMIM 185535), *APC* (OMIM 611731), *AXIN2* (OMIM 604025), *BMPR1A* (OMIM 601299), *CDK4* (OMIM 123829), *CDKN2A* (OMIM 600160), *ERCC2* (OMIM 278730), *FANCC* (OMIM 613899), *MSH3* (OMIM 600887), *MUTYH* (OMIM 604933), *NTHL1* (OMIM 602656), *POLD1* (OMIM 174761), *POLE* (OMIM 174762), *PTEN* (OMIM 601728), *SMAD4* (OMIM 600993), *VHL* (OMIM 608537).

### Reporting summary

Further information on research design is available in the Nature Research Reporting Summary linked to this article.

### Supplementary information

REPORTING SUMMARY

Supplementary

## Data Availability

The datasets used and/or analyzed during the current study are available from the corresponding author through collaboration and/or data usage agreement under the Genomics Thailand data protection and usage regulation by the Health Systems Research Institute of Thailand.

## Code availability

Torrent Suite™ Software (Thermo Fisher Scientific, USA) was used for primary data analysis. SEQUENCE Pilot—SeqNext (JSI Medical Systems GmbH, Germany) was used for base calling, re-alignment, variant calling, and annotation. VarSeq—VSClinical software (Golden Helix, USA) was used for variant analysis and classification. No custom code was used to generate or process the data described in the manuscript.
