# Supplementary material for: Germline mutations of 4567 patients with hereditary breast-ovarian cancer spectrum in Thailand
Source: NPJ Genom Med. 2024 Feb 14;9:9. doi: 10.1038/s41525-024-00400-4 (PMC10866978; doi:10.1038/s41525-024-00400-4)
Supplement: Supplementary file 2 — Supplementary [file 41525_2024_400_MOESM2_ESM.pdf]

Supplementary Table 1: Non-HBOC-related cancers in patients with P/LP variants in hereditary breast-ovarian cancer

Genes	Colorectal	Endometrium	Biliary tract	Sarcoma	Brain (CNS)	Renal & urothelial	Stomach	Hemato-logic	Lung
BRCA1	3	4		1			1		
BRCA2	3	1							1
TP53		1		2	2	1		1	
ATM	4	1							
CDH1	1								
PTEN		1				1			
BARD1		1	1						1
RAD50		1					1		
MLH1									
MSH2	2	1	1			1			
PMS2				1					
Total	13	11	2	4	2	3	1	1	1

Supplementary Table 2: Available clinical data for patients with P/LP variants in cancer susceptibility genes who did not meet current NCCN criteria

Clinical features	n
Clinical histories favored the likelihood of hereditary cancer but did not meet the written criteria for genetic testing	19
Family history of cancer not indicated in the guidelines<br>- First-degree relatives with breast cancer at an age older than 50 years<br>- Other types of cancer beyond the HBOC spectrum (colon, endometrium, lung, brain, kidney, liver, cervix, sarcoma)	16
Second primary cancer besides breast, ovarian, pancreatic, or prostate (including colorectal cancer, endometrial cancer, lung cancer, sarcoma)	8

Supplementary Table 3: Clinical features of ovarian cancer patients who had P/LP variants in mismatch repair (MMR) genes (Lynch syndrome; LS)

**Clinical features**	
Median age of ovarian cancer diagnosis (years)	41 (IQR 15.5)
**Available histopathologic results**	**n = 5**
- Clear cell carcinoma	2
- Endometrioid carcinoma	1
- Mucinous cystadenocarcinoma	1
- Signet ring cell carcinoma	1
**Clinical history available**	**n=11**
• **Multiple primary LS-associated cancer**	**n = 4**
- Colorectal cancer	2
- Endometrial cancer	1
- Urothelial cancer	1
• **Family history of LS-associated cancer**	**n = 2**
- Colorectal cancer	1
- Endometrial cancer	1

Supplementary 4: Method - DNA extraction, library preparation, sequencing platform and data analysis for genetic testing

Peripheral blood specimens from each patient were collected to extract genomic DNA using the magnetic-based Chemagic™ DNA Blood Kit (PerkinElmer, USA) following the manufacturer's protocol. The extracted DNA was quantified using a NanoDrop 1000 Spectrophotometer and a Qubit assay (Thermo Fisher Scientific, USA). Working DNA, with a concentration of 10 ng/µL, was used for library preparation, employing the Ion AmpliSeq Library preparation kit along with custom-tailored primer pools, covering the entire coding regions and splice junctions of the genes. Subsequently, DNA sequencing was carried out on the Ion S5 XL system using the Ion Torrent™ Ion 540™ Kit-Chef and Ion 540 chip kit. Primary NGS data analysis, including base calling and read alignment, was performed using Torrent Suite™ Software (Thermo Fisher Scientific, USA). The Binary Alignment Map (BAM) file was further analyzed for base calling, re-alignment, variant calling, and annotation using SEQUENCE Pilot – SeqNext (JSI medical systems GmbH, Germany). The analysis was conducted with the following settings: base calling within the region of interest (ROI) required a minimum coverage of 30x, and variant calling required a minimum coverage of 15x, with a minimum variant allele fraction of 15% for both forward and reverse sense strands. For variant analysis and classification, the variant call format (VCF) and BAM files were transferred to VarSeq – VSClinical software (Golden Helix, USA).
